# Sexual assault in Lagos, Nigeria: a five year retrospective review

**DOI:** 10.1186/1472-6874-14-115

**Published:** 2014-09-23

**Authors:** Fatimat M Akinlusi, Kabiru A Rabiu, Tawa A Olawepo, Adeniyi A Adewunmi, Tawaqualit A Ottun, Oluwarotimi I Akinola

**Affiliations:** Department of Obstetrics and Gynaecology, Lagos State University College of Medicine, Ikeja, Lagos, Nigeria

**Keywords:** Rape, Sexual assault, Violence against women, Survivors

## Abstract

**Background:**

Cases of sexual assault are increasingly reported. However, Nigerian researchers have not given adequate attention to this subject despite its attendant social, physical and psychological consequences.

This study assessed survivors’ characteristics, circumstances of assault and treatment offered with a view to reducing the incidence as well as improving evaluation and management.

**Methods:**

A retrospective review of survivors’ case records at Lagos State University Teaching Hospital, Ikeja, between January 2008 and December 2012. Data was analysed using the Epi-info 3.5 statistical software of the Centre for Disease Control and Prevention, Atlanta U S A.

**Results:**

Of the 39,770 new gynaecological cases during this period, 304 were alleged sexual assault giving an incidence of 0.76% among hospital gynaecological consultations. Only 287 case notes had sufficient information for statistical analysis. Of these, 83.6% were below 19 years, 73.1% knew their assailants (majority were neighbours), most assaults (54.6%) occurred in the neighbours’ homes and over 60% of victims presented after 24 hours of assault.

Although 77.3% were assaulted at daytime, teenagers were likely to be raped during the day and non-teenagers at night (P < 0.001). Threat and physical violence were mostly used to overcome victims. Seventy three point six percent had Human Immunodeficiency Virus (HIV) screening with one positive at onset. Post Exposure Prophylaxis for HIV was given in 29.4% of those eligible and emergency contraception in 22.4% of post-menarcheal victims (n = 125).

There were neither referrals for psychotherapy nor forensic specimen collected. No record of post-assault conception or HIV infection was found during follow-up.

**Conclusions:**

Adolescents remain the most vulnerable requiring life skills training for protection. Survivors delay in presenting for care. Therefore, public enlightenment on the benefits of early interventions and comprehensive care of survivors with the use of standardized protocols are recommended.

## Background

Sexual assault is a severely traumatic experience that disproportionally affects adolescent and young adult women [1] and is often associated with psychological, physical and social distress [2].

Though researchers’ definition of sexual assault varies [3], it includes a spectrum of activities ranging from rape to physically less intrusive sexual contacts, whether attempted or completed [3].

Rape is not a medical diagnosis. It is a legal terminology reserved for cases of penile penetration of the victim’s vagina, mouth, or anus without consent [4]. Other types of sexual assault include forced or coerced vaginal or anal penetration by any other body parts or object; breast or genitalia fondling; or being forced or coerced to touch another person’s genitalia [5]. It involves lack of consent; the use of physical force, coercion, deception or threat; and/or the involvement of a victim that is mentally incapacitated or physically impaired (due to voluntary or involuntary alcohol or drug consumption), asleep or unconscious [6].

Sexual assault is not peculiar to any particular race or socio-economic class. The World Health Organization reports that one in every five women is a victim of sexual assault [7] and globally, 35% of women have experienced either physical and/or sexual intimate partner violence or non-partner sexual violence [8]. The regions of the world with the highest reported rates of sexual and physical violence towards women are Africa, the Middle East and Southeast Asia [8].

In Africa, 5–15% of the females report a forced or coerced sexual experience [9]. In South Africa, prevalence of rape, from community based reports show a figure of 2070 per 100,000 per year [10]. Reports from Ethiopia showed from a study of 367 high school girls, that 11.4% of them had started having intercourse and 33.3% of this group was rape [11]. Adolescents however have the highest rates of rape and other sexual assaults of any age group [12].

In Nigeria, the reported incidence of sexual assault varies depending on study design and methodology. A study in Ibadan showed that 15% of young females reported forced penetrative sexual experience [13], while 13.8% prevalence rate was found in female Maiduguri students [14]. True incidences are inaccurate and often underestimated since most cases of sexual assault are under-reported by the victims because of the associated stigma [14].

The violence involved in an attempted sexual assault can have the same impact on the survivor as a completed one. The impact can be immediate or delayed with long-term health consequences for survivors. Significant social and economic consequences also occur. Health consequences include physical injuries, unwanted pregnancies, unsafe abortions and sexually transmitted diseases, including HIV. Immediate psychological reactions such as shock, shame, guilt and anger may be exhibited [15] while long-term psychological outcome include depression, post-traumatic stress disorder, suicidal ideation, lack of sexual enjoyment, and fear [15]. A recent systematic review found that women who have been sexually assaulted by non-partners are 2.3 times more likely to use alcohol and 2.6 times more likely to experience depression or anxiety while those abused by partners are 1.5 times more likely to have a sexually transmitted disease, including HIV [8].

Adolescents are particularly susceptible to HIV transmission through forced and unforced sex because their vaginal mucous membranes have not yet acquired cellular density significant to provide an effective barrier that develop in later teenage years [7].

In Nigeria, cases of sexual assault are increasingly reported. Lagos, like other megacities world over predisposes its women population to higher risks of sexual violence [16] amongst other crimes. Despite this, many Nigerian researchers have not given adequate attention to this subject especially in terms of the evaluation of care given to survivors.

At the Lagos State University Teaching Hospital (LASUTH), Ikeja, no review has yet been done on sexual assault. This has therefore necessitated a review of these cases with the aim of determining survivors’ characteristics, circumstances of assault and treatment offered with a view to reducing the overall incidence as well as improving survivors’ evaluation and management.

## Methods

### Design

This is a retrospective descriptive study.

### Setting

The study was carried out at the Lagos State University Teaching Hospital (LASUTH), Ikeja. Patients come from within and outside Lagos and an average of 12 new gynaecological patients is seen every day of the week [17].

### Study population

Survivors of alleged sexual assault who self- presented to Lagos State University Teaching Hospital (LASUTH), Ikeja during the study period of January 2008 to December 2012 were the subjects of review. The hospital has a 20-bedded gynaecological ward that will soon be replaced by a substantive facility nearing completion. The staff population is a mixture of 11 specialist gynaecologists, variable number of resident doctors, nurses and other support staff.

The medical records department was approached for the identification of case notes of all females that self- presented to LASUTH for medical care within the study period having experienced any form of sexual assault.

A sexual assault case was defined as any person, irrespective of age reporting any type of non-consensual sexual activity whether attempted or completed.

The hospital does not have a written protocol for the management of cases of sexual assault.

However, when a victim of sexual assault presents at the emergency room, the triage nurse will perform the initial assessment, including vital signs. Consultation is then arranged with the duty registrar who identifies emergent needs, provides comfort and explains services. Subsequent evaluation will include obtaining detailed history, conducting a thorough physical examination, obtaining forensic evidence if indicated, as well as testing for sexually transmitted infections, pregnancy and HIV. Comprehensive treatment includes treatment of injury, provision of emergency contraception, prophylactic antibiotics for sexually transmitted infections, tetanus and hepatitis B vaccination and post-exposure prophylaxis for HIV. Appropriate documentation of physical findings and care provided are made. Counselling and psychosocial support are also offered. The client is supported if she decides to involve police or legal authority.

Of recent, a non-governmental sexual assault response centre, MIRABEL CENTRE now operates within LASUTH. This centre also provides acute care and long term follow of survivors.

### Data collection

The case records of survivors were retrieved, reviewed and information extracted was entered into a proforma that evaluated socio-demographic characteristics, place and time of the incident, relationship of assailants to victims, methods employed by assailant to overcome victims, forensic specimen collection, treatment offered and follow up of survivors.

### Analysis

Categorization of open-ended questions was done and data obtained was entered into the computer and analysed using Epi-info statistical software (2008 version). These were then presented in tabular and descriptive forms. Bivariate (inferential) analysis was also done.

Chi square test was used to compare proportions between variables where appropriate, at 95% confidence level. A p-value of less than 0.05 was considered to be statistically significant.

### Ethics

Ethical approval was given by the research and ethics Committee of the Lagos State University Teaching Hospital.

Confidentiality was maintained by omitting survivors’ names from the proforma.

Written informed consent was not obtained from participants as this is a retrospective study.

## Results

During the study period, there were 39,770 Gynaecological consultations, 304 of which were for alleged sexual assault. Only 287 of these had sufficient information for statistical analysis. The incidence of those reporting to the hospital with suspected or confirmed sexual assault among hospital gynaecological consultations was thus 0.76% for the period under review.

**Figure 1:**
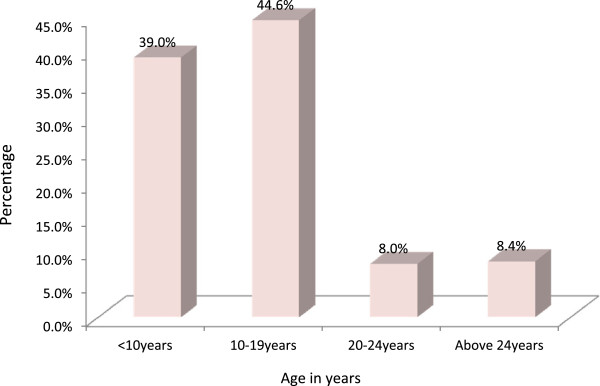
**Age distribution of victims.** Adolescents (age group 10–19) accounted for the majority (44.6%) of the cases, followed by children less than 10 years (39.0%).

Table 1 shows the socio-demographic characteristics of assaulted victims. The ages ranged from 2 to 50 years with a mean of 12.9 ± 8.758 years. Most victims were of Christian faith and Yoruba ethnic group and majority lived with their parents (70.7%). Adolescents (age group 10–19) accounted for the majority (44.6%) of the cases, followed by children less than 10 years (39.0%), making the entire under 19 age group 83.6% of all victims. This is also depicted in Figure 1.

**Table 1:** **Socio-demographic characteristics of assaulted victims**

Characteristic	Frequency	Percentage (%)
**Age group**		
<10	112	39.0
10-19	128	44.6
20-24	23	8.0
>24	24	8.4
**Occupation**		
Student	213	74.2
Pre- school	18	6.3
Apprentice	17	5.9
Professional	16	5.6
Domestic servant	12	4.2
Unemployed	6	2.1
Hawker	5	1.7
**Religion**		
Christianity	223	77.7
Islam	64	22.3
**Tribe**		
Yoruba	151	52.6
Igbo	87	30.3
Others	45	15.7
Hausas	4	1.4

Students accounted for 74.2% of the occupation cadre and 40.1% of them were assaulted in neighbors’ houses but no assault occurred in the school. Pre-pubertal victims constituted 56.4% while 43.6% were post pubertal.

Although 77.3% of all victims were assaulted at daytime and 22.7% at night, as many as 88.9% of victims raped during the day were less than 19 years. Teenagers and young girls were more likely to be raped during the day than at night when compared to older adults (P < 0.001). This is depicted in Table 2. The chance of a teenager experiencing daytime sexual assault was more than 17 times higher than that of an older adult.

**Table 2:** **Comparison of age versus time of incidence**

Characteristics	Day time	Night	OR	CI	P-value
Teenagers (<20 year)	208 (87.0%)	31 (13.0%)	17.548	8.354 -36.862	<0.001
Non-teenagers (>20)	13 (27.7%)	34 (72.3%)	0.057	0.027 - 0.120	<0.001

Sixty four point five percent (64.5%) of the victims presented after 24 hours of assault as shown in Table 3. The assailants were well known to the victims in 73.1% of cases and were mostly neighbours. A description of the place of assault is presented in Table 4 where the neighbors’ home accounted for 54.6%.

**Table 3:** **Disclosure interval and relationship of the assailants to victims**

Characteristics	Frequency	Percentage (%)
**Disclosure interval**		
Within 24 hours	102	35.5
24 hours – 6 days	129	45.0
7 days – 1 month	31	10.8
>1 month	25	8.7
**Assailants**		
Neighbours	157	54.9
Stranger	77	26.9
Acquaintances	36	12.6
Family member	13	4.6
Boy friend	3	1.0

**Table 4:** **Place of assault and management of assaulted victims**

Characteristics	Frequency	Percentage (%)
**Place of assault**		
Neighbour’s house	156	54.6
Victim’s home	60	21.0
Uncompleted building	34	11.9
Street corner	27	9.4
Friend’s house	9	3.1
**Treatment**		
HIV screening	209	73.6
Follow up (once)	204	72.3
HVS	181	63.7
HBsAg screening	143	50.5
VDRL Testing	131	46.2
Pregnancy test	86	30.5
PEPs	72	25.4
Emergency contraceptives	28	9.7

Methods of overcoming the victims included threat (35.5%), deceit (24.1%), physical violence (28.7%), money (9.8%) or alcohol (2.1%). Thirty six point one percent (104) of the victims reported previous sexual exposure. The total number of assailants involved at different instances ranged from 1 to 9.

The management offered is summarized in Table 4. Seventy three point six percent (73.6%) had Human Immunodeficiency Virus (HIV) screening with one positive at onset. Post Exposure Prophylaxis for HIV was given in 29.4% of those eligible and emergency contraception in 22.4% of post-menarcheal victims (n = 125).

The commonest isolated organism was *candida species* (13.1%). There were neither referrals for psychotherapy nor forensic specimen collected. No record of post-assault conception or HIV infection was found during follow-up and more than a quarter of the victims (27.7%) did not return for follow-up.

## Discussion

This is a 5 year retrospective study of cases of sexual assault in LASUTH between January 2008 and December 2012. The incidence of sexual assault of 0.76% is low compared to similar studies in Nigeria where incidences of 2.1%, 5.6%, 7.7% and 13.8% were reported in Calabar, Jos, Benin and Maiduguri respectively [9, 14, 18, 19]. This low figure is probably due to the very large denominator of 39,770 new gynaecological consultations during the study period. LASUTH attends to referred patients from several General and Private Hospitals in the state and neighbouring states.

An age range of 2 to 50 years was found in this study. This is in contrast to the findings in Calabar and Benin, Nigeria with 4 to 23 and 3 to 25 years respectively. An Indian study of victims of sexual assault however reported a similar range of 3 and 42 year [20]. This disparity in the maximum age of victims may be due to underreporting to police and health authorities by the older survivors who may fear loss of societal respect especially in the traditional and less metropolitan cities of Calabar and Benin.

In this study, girls less than 19 years accounted for 83.6% of cases seen. This is comparable with other surveys [9, 19, 21], where a disproportionate number of sexual assaults occurred among children and adolescents.

Children 10 years and below contributed almost 40% of this vulnerable age group in this study. They tend to offer little or no resistance to their assailants. Additionally, inadequate parental care may be more prevalent in the Lagos setting where high cost of house rents prevents accommodation near work places. The heavy traffic situation further delays the return of parents and guardians to their homes. These children may have to be left to the care of neighbours or in some other places where supervision is inadequate. These expose them to potential abuse by minders who may even use them as errand girls. The above may explain why most assaults (77.3%) occurred during the daytime since parents are away from home at this period.

In 73.1% of cases, the victims knew their assailant. This is documented in studies elsewhere where the perpetrators of the sexual assaults were blood relations, neighbours, acquaintances, authority figure and stranger [9, 19, 21, 22]. Neighbours were assailants in 54.9% of cases which explains why 54.6% of rape occurred in neighbours’ homes where the possibility of being caught in the act is quite slim. In the Jos study however, most assaults (46.6%) occurred in the victim’s home.

The time interval between the alleged rape and disclosure varied widely from less than 24 hours to three months. In 35.5% of victims, reports were made within 24 hours while most (45%) reported between 24 hours and 6 days of the incident. The wide variation in the interval of disclosure could be attributed to threats of violence or death which has been found in this study to be the most common means of subduing the victims. Children particularly believe assailants’ threats and would not report until parents discover; some fear they may not be believed [23]. For the older victims, the fear of stigmatization could be responsible for delayed disclosure [23].

The longer the interval, the lower the quantity and quality of forensic evidences [24], and the higher the risk of negative health outcomes.

Ninety two point two percent of the victims had made reports to the police prior to presentation. This may suggest that victims are more interested in having their assailants punished than for their own medical care. An official report to the police will however mandate a hospital assessment and report which ensures appropriate medical, legal and psychological actions [23].

Few of the victims had body abrasions (9.4%). It may be that the late presentation had allowed for healing while submission of the victim may be achieved by emotional manipulation or verbal threats leaving no injuries.

The standard of clinical management of sexual violence involves documentation and treatment of injury, getting forensic materials, detecting prior pregnancy, screening for sexually transmitted infections including HIV and provision of adequate contraception, post exposure prophylaxis [24] and supportive psychosocial counselling.

High vaginal swab testing and HIV screening were done in 63.7% and 73.6% of cases respectively while just about half of the victims were screened for Hepatitis B surface Antigen (HBsAg) and Venereal Disease Research Laboratory Test (VDRL).

Of the 201 victims who presented within 72 hours of assault, only 59 (29.4%) were referred for post exposure prophylaxis of HIV (PEP) at the Haematology Department despite the importance of HIV prevention. PEP might have been withheld in survivors with apparent low risk of HIV transmission from the assault. The recommendation is that post exposure prophylaxis be provided to sexual assault victims especially when there is mucosal exposure; trauma and bleeding; in cases of repeated sexual abuse or multiple perpetrators; when the assailant is known to be HIV positive or has high risk behavior for HIV infection; or in places of high HIV prevalence [25].

Only 55.7% of the post-pubertal victims had pregnancy test done and just 22.4% of same group had emergency contraceptives. It was found that the nature of the assault and disclosure interval contributed to this low proportion. This is in keeping with other studies where emergency contraception to prevent post rape pregnancy was not consistently offered to rape victims [26].

This review revealed that no forensic samples were collected, neither were there referrals for psychotherapy. The lack of forensic evidence will no doubt hinder justice, encourage perpetuation of rape and promote non-disclosure. Since psychological consequences may occur in the acute period or in the long run and psychotherapy is a recognized way of moderating the negative effects on victims, this should be offered in all cases.

A heightened risk for sexual victimization among adolescents and young girls consistent with other studies is further supported here and this underscores the need for prevention and intervention efforts targeted at this population. There is a need to encourage organizations and policy makers to create programs to prevent sexual assault in this vulnerable population. Such will include age-appropriate sexual assault education which will not only help in reducing risk for sexual assault, but also improve chances that an assault will be reported when it occurs.

Of note is the fact that most cases are not reported early which translates to delay in seeking care. Since the very low incidence may also suggest gross underreporting, it is necessary to incorporate a thorough violence/assault assessment into routine history-taking procedures for non-sexual assault-related consultations. This will help increase the disclosure of any previous sexual assaults.

Survivors want justice as evidenced by the proportion of those who had made police reports. The judicial system will need to be strengthened to handle assault cases effectively as this encourages formal reporting and helps to hold perpetrators accountable while deterring like-minded individuals from committing similar crimes.

Regular in-service training of health care providers and the utilization of written guidelines for the management of sexually assaulted victims will prevent omissions and ensure prompt and comprehensive post-rape care.

The hospital-based nature of this study limits the generalizability of its findings to the larger population. Being a retrospective review, this study was also constrained by the availability of data in the case records.

## Conclusions

Sexual assault is a grievous under reported offence with detrimental physical, social and psychological effects on its victims. Adolescents continue to have the highest rates of all age groups. Assailants are often known to their victims who perpetrate this act during the daytime. Survivors delay in seeking care. There is a need for a more comprehensive evaluation and care of victims in order to prevent serious sequelae and deter assailants. This may be achieved by the utilization of standardized protocols.

Increased public awareness and preventive interventions are required particularly within the at-risk age group to enhance their safety.

## Abbreviations

HIV: Human Immunodeficiency Virus
LASUTH: Lagos State University Teaching Hospital
HBsAg: Hepatitis B surface Antigen
VDRL: Venereal Disease Research Laboratory Test
PEP: Post exposure prophylaxis of HIV
HVS: High Vaginal Swab.
